# Infrastructure-Free Indoor Pedestrian Tracking with Smartphone Acoustic-Based Enhancement

**DOI:** 10.3390/s19112458

**Published:** 2019-05-29

**Authors:** Chao Liu, Sining Jiang, Shuo Zhao, Zhongwen Guo

**Affiliations:** Department of Information Science and Engineering, Ocean University of China, Qingdao 266100, China; jsn@stu.ouc.edu.cn (S.J.); snickerfish9@gmail.com (S.Z.); guozhw@ouc.edu.cn (Z.G.)

**Keywords:** infrastructure-free indoor pedestrian tracking, acoustic signal, Doppler effect, Inertial Measurement Unit, arbitrary wandering

## Abstract

Indoor pedestrian tracking has been identified as a key technology for indoor location-based services such as emergency locating, advertising, and gaming. However, existing smartphone-based approaches to pedestrian tracking in indoor environments have various limitations including a high cost of infrastructure constructing, labor-intensive fingerprint collection, and a vulnerability to moving obstacles. Moreover, our empirical study reveals that the accuracy of indoor locations estimated by a smartphone Inertial Measurement Unit (IMU) decreases severely when the pedestrian is arbitrarily wandering with an unstable speed. To improve the indoor tracking performance by enhancing the location estimation accuracy, we exploit smartphone-based acoustic techniques and propose an infrastructure-free indoor pedestrian tracking approach, called iIPT. The novelty of iIPT lies in the pedestrian speed reliability metric, which characterizes the reliability of the pedestrian speed provided by the smartphone IMU, and in a speed enhancing method, where we adjust a relatively less reliable pedestrian speed to the more reliable speed of a passing by “enhancer” based on the acoustic Doppler effect. iIPT thus changes the encountered pedestrians from an“obstacle” into an “enhancer.” Extensive real-world experiments in indoor scenarios have been conducted to verify the feasibility of realizing the acoustic Doppler effect between smartphones and to identify the applicable acoustic frequency range and transmission distance while reducing battery consumption. The experiment results demonstrate that iIPT can largely improve the tracking accuracy and decrease the average error compared with a conventional IMU-based method.

## 1. Introduction

Considered as the last step of mobile service, indoor pedestrian tracking (IPT) has been widely utilized by researchers for the study of indoor location-based applications and services. For example, shopping mall navigation provides convenient shopping guidance and marketing services for customers. Hospitals leverage IPT systems to locate their specific patients in case of sudden accidents. Without IPT applications, people can get lost when they are trying to find their car in a large parking lot. Due to the building materials, GPS is unavailable for IPT, leading to a constant emerging of various smartphone-based tracking methods that have the capability of obtaining location information via Wi-Fi [1], Bluetooth [2,3], LED [4,5], and other techniques.

Each smartphone-based IPT method has its pros and cons, and how to develop a low-cost, reliable, and accurate IPT system is still an outstanding research problem. Many attempts have been made in recent years. Yin et al. [6] utilized radio and visual features of a diagram of Wi-Fi fingerprints to realize IPT, but this still requires intensive labor for fingerprint collection. By leveraging additional beacons, Xiang et al. [7] combined Bluetooth and crowdsourcing to estimate pedestrian locations. In [8], a generic indoor localization framework is proposed based on existing lighting infrastructure, which is sensitive to moving obstacles. Limited by the demand for extra infrastructures, these methods suffer a relatively high cost. In order to realize infrastructure-free IPT, researchers leverage smartphone in-built sensors, such as inertial measuring units (IMUs), to estimate a pedestrian’s location. IMUs in commercial smartphones have been widely utilized for pedestrian dead-reckoning (PDR) [9], which contain multiple sensors such as accelerometers, gyroscopes, and sometimes magnetometers, and are capable of tracking the movement of pedestrians by detecting steps, estimating stride lengths, and the directions of motion. However, the tracking accuracy of PDR decreases severely when the pedestrian walks around arbitrarily, causing accumulative error. To improve PDR accuracy, researchers [10,11] focus on the enhancement of a pedestrian’s steps detection and stride length estimation, but they still cannot satisfy the actual accuracy requirement due to the complex indoor environment and the pedestrian’s arbitrary walking. Recently, acoustic-based IPT [12] has shown its unique advantages of accurate positioning and direction finding. To be more specific, ultrasonic-based IPT systems [13,14] perform well at estimating location with a high degree of accuracy. Ultrasonic frequency is normally 20–44 kHz (inaudible for human) and easily removes noise. Due to the low velocity of ultrasonic wave traveling in air, high accuracy time of flight (ToF) measurement is allowed. Moreover, the Doppler effect has been widely utilized to track mobile objects [15,16]. However, there are two main limitations that affect the performance of ultrasonic-based IPT. Firstly, similar to other smartphone-based IPT methods, extra infrastructures and self-designed devices are required to guarantee the tracking accuracy by emitting and receiving ultrasonic signals, which is time-consuming and has a high cost. Secondly, ultrasonic signals can be easily blocked by mobile obstacles due to its physical features, causing reflection and multi-path problems, ultimately decreasing tracking accuracy. According to [17], smartphones can emit and receive ultrasonic signals between each other. What if we utilized smartphones only to accomplish IPT by combining the advantages of ultrasonic and IMU methods?

In this paper, we propose iIPT, an infrastructure-free pedestrian tracking approach, by combining smartphone-based acoustic and IMU techniques. A metric called pedestrian speed reliability is presented to characterize the reliability of the pedestrian speed provided by the smartphone IMU. We adjust a relatively less reliable pedestrian speed to a more reliable speed of a passing by ”enhancer“ based on the acoustic Doppler effect, thus changing the encountered pedestrians from an “obstacle“ into an ”enhancer“. Extensive real-world experiments in indoor scenarios have been conducted to verify the feasibility of realizing the acoustic Doppler effect between smartphones and to identify the applicable acoustic frequency range and transmission distance while reducing battery consumption. The experiment results demonstrate that iIPT can largely improve the tracking accuracy and decrease the average error compared with a conventional IMU-based method. We have made several contributions as follows:

- We present an infrastructure-free indoor pedestrian tracking approach by combining both smartphone-based acoustic and IMU techniques.
- We present a pedestrian speed reliability metric that characterizes the reliability of the real-time pedestrian speed provided by smartphone IMU and reflects the arbitrariness of the pedestrian walking pattern.
- We determine the capability and precision of measuring speed using sonic Doppler from a smartphone by designing a robot car whose speed could be controlled to compare the real speed.
- We leverage the acoustic Doppler effect to adjust the relatively less reliable pedestrian speed to a more reliable speed of a passing by “enhancer” measured by IMU.
- We implement comprehensive experiments to identify the applicable acoustic frequency range, transmission distance, and battery consumption and demonstrate that iIPT can largely improve the tracking accuracy and decrease the average error, compared with PDR.

The remainder of this paper is organized as follows: Section 2 illustrates our motivation and challenges for proposing iIPT. In Section 3, an overview of iIPT is presented and Section 4 elaborates our indoor pedestrian tracking approach. Section 5 evaluates the performance of iIPT in real situations. In Section 6, we discuss about the limitations and unsolved problems for iIPT. Finally, Section 7 provides a brief conclusion.

## 2. Motivation and Challenges

In a common indoor environment, a pedestrian’s attention can be easily drawn by many places of interest, leading to different walking patterns. For indoor pedestrian tracking with smartphone IMUs, a sudden stop or speed up will increase the complexity of walking detection and lower the tracking accuracy. In this scenario, we present two important observations.

**Observation 1.** A pedestrian usually walks arbitrarily with an unstable speed in an indoor environment. According to research [18,19] on human mobility models in indoor environments, pedestrian mobility can be influenced by “social forces,” including certain motion requirements, other, disturbing pedestrians, and various attractive effects. For example, customers are commonly attracted by clothing shops or restaurants when shopping in a mall. Visitors occasionally concentrate on gorgeous art work at exhibitions when they have great interest in it. These kinds of cases happen quite often in indoor environments and cause problems for IMU-based indoor pedestrian tracking, such as PDR, a common IPT method that is capable of tracking pedestrians by estimating their stride frequency, stride length, and walking direction.

**Figure 1:**
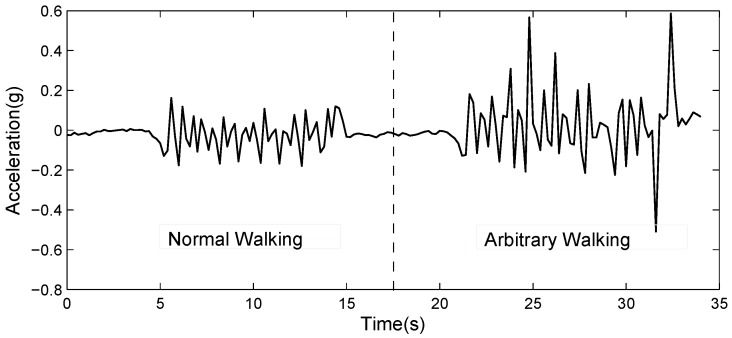
A value change of acceleration sensed by smartphone inertial measuring units (IMUs) under different pedestrian walking patterns.

**Figure 2:**
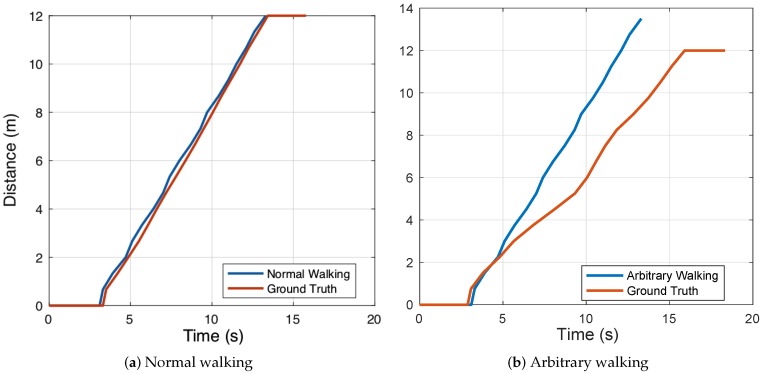
Accumulative distance bias caused by pedestrian dead-reckoning (PDR) with different pedestrian walking patterns.

**Observation 2**. The indoor pedestrian tracking accuracy of the IMU-based method decreases severely when a pedestrian walks arbitrarily. As shown in Figure 1, when a pedestrian walks normally with uniform speed, the value of acceleration sensed by accelerometers follows a regular pattern so that a pedestrian’s stride frequency can be easily obtained and we can directly utilize their average stride length to estimate the walking distance. The result (see Figure 2a) depicts that PDR performs well in tracking a pedestrian with arbitrary walking. The ground truth represents the pedestrian’s actual walking distance, which is obtained by a manual method. After making fixed marks on the ground, we utilize a stopwatch to record the time that a pedestrian steps on the marks. However, Figure 2b shows that the accumulative distance bias of PDR keeps increasing if we utilize a conventional PDR method to analyze the acceleration data caused by arbitrary walking. The estimated stride frequency will be larger than the actual value, and the average stride length will not make any sense. Some researchers [10,11] have used a machine learning algorithm to optimize PDR, but these methods require plenty of pedestrian history trajectory as training data and the PDR accuracy relies too much on the quality of the training result.

Based on our observations mentioned above, we have done relevant research, as follows.

**Indoor Pedestrian Dead-Reckoning**. IMU is widely utilized in scenarios of indoor PDR. Jimenez et al. [20] proposed a self-designed IMU attached to the foot of a person for pedestrian tracking, leveraging accelerometers, gyroscopes, and magnetometers to detect step detection, stride length, and heading. Other researchers leverage smartphone-based IMUs to accomplish PDR. In [10,11], researchers use a machine learning algorithm to optimize PDR, but these methods require plenty of pedestrian history trajectory as training data, and the PDR accuracy relies too much on the quality of the training result. Other attempts [21,22,23] have also been made to improve PDR accuracy. However, how to improve its accuracy is still an open issue that needs to be addressed.

**Ultrasonic Applications on Smartphones**. Basic research [24] has been made to evaluate the innate ability of mobile phone speakers to produce ultrasonic signals under different volume levels, but the ultrasonic emit/receive ability, the transmission distance, and the battery consumption of smartphones are not mentioned. A few researchers have taken advantage of ultrasonic physical features to accomplish high precision measurements. Peng et al. [25] designed a high-accuracy acoustic-based ranging system, which can be easily applied to smartphones and relies on the TOA and TDOA methods to calculate distance. Other researchers [26,27,28,29] are working on indoor pedestrian or robot localization and tracking at long distance (more than 20 m) by leveraging ultrasonic signals with smartphones. However, most of them require a self-designed ultrasonic emitter or receiver array based on their own demand, which is time-consuming and has a high cost. Moreover, the tracking accuracy can be easily influenced by obstacles between the emitter and the receiver. In a word, all these methods cannot be directly utilized in our scenario.

**Ultrasonic Doppler Effect**. The Doppler effect has been widely utilized in many scenarios of mobile object tracking with smartphones. Wang et al. [15] proposed a device-free method to track gestures of a hand/finger. Based on the Doppler effect, they analyzed the acoustic phase to obtain movement direction and distance measurements. In order to provide an enjoyable user experience, Mao et al. [30] developed a high-precision acoustic tracker to replace a traditional mouse and allow a user to play VR games by moving a smartphone in the air. However, both of these are operated at short distances (from 1 cm to 2 m). Similar to our scenario, DopEnc [17] is an acoustic-based encounter profiling system on smartphones, using the Doppler effect method to calculate relative speed and build an encounter profile. Swadloon [12] performs an accurate acoustic direction finding scheme, according to the arbitrary pattern of phone shaking in a rough horizontal plane.

We are motivated to deal with pedestrian arbitrary walking with unstable speeds and to leverage the smartphone acoustic Doppler effect to enhance the tracking accuracy of the IMU-based method. In this paper, we consider that the acoustic signals with frequencies from 17 to 20 kHz is extremely similar to ultrasonic signals and thus call it sub-ultrasonic, which represents the particular acoustic signals we apply throughout the paper. The elaboration of sub-ultrasonic signals is shown as follows.

Ultrasonic has been widely utilized in high accuracy detection and measurement. However, according to our experiment result (see Table 1), commercial smartphones can only emit and receive acoustic less than 20 kHz, and more than 17 kHz is inaudible for young babies and other, sensitive people. More detail is illustrated in Section 4.2.

In the next section, we present an overview of iIPT.

## 3. Overview

In this section, we first present the basic idea for iIPT and then give a brief introduction for IMU-based PDR and the acoustic-based Doppler effect. Moreover, we identify three key issues for iIPT, which will be addressed in Section 3.4.

### 3.1. Basic Idea

**Figure 3:**
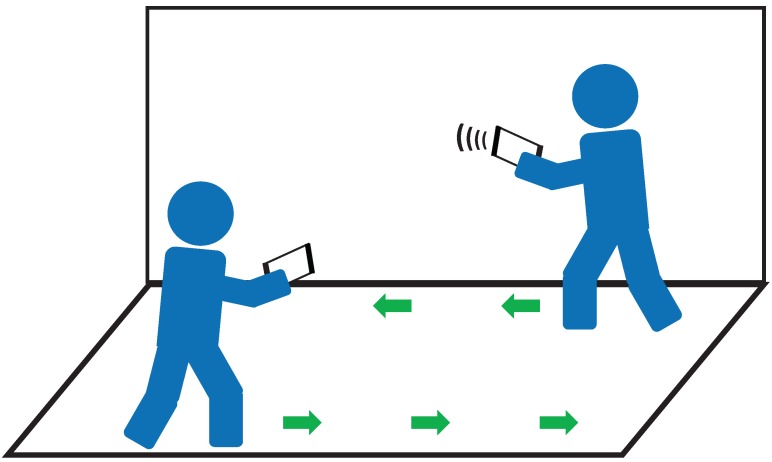
Indoor pedestrian encounter model for infrastructure-free indoor pedestrian tracking (iIPT).

Unlike existing ultrasonic IPT systems, iIPT requires no extra infrastructure and only utilizes smartphones to improve tracking accuracy. In our indoor scenario (see Figure 3), the pedestrians are separated into two groups: users and encounters. In most cases, the encounter will be treated as an “obstacle” who has the possibility of decreasing the tracking accuracy. For example, encounters nearby may change the received signal strength indication (RSSI) so that Wi-Fi signal fingerprints becomes unreliable. In quite the opposite, we take the most advantage of encounters and consider them as an “enhancer” for iIPT instead.

When the user walks arbitrarily with unstable speed, IMU data becomes unreliable, leading to accumulative distance bias by PDR. The user discontinuously emits a sub-ultrasonic signal, and once an encounter with more reliable IMU data receives the signal, iIPT begins to calculate their relative speed based on the sub-ultrasonic Doppler effect. The user’s absolute speed will be obtained by taking both the relative speed and the encounter’s speed from IMU into account. Finally, the user’s walking distance can be estimated by integrating their walking speed during a certain period. Due to the sub-ultrasonic physical feature (straight line traveling and low penetration), we only focus on the face-to-face pedestrian encounter scenario in a hallway-like indoor space.

### 3.2. Shortcomings of PDR

IMU-based PDR plays a vital role in indoor pedestrian tracking. Embedded in smartphones, accelerometers and gyroscopes can detect pedestrian stride frequency and heading direction so that walking trajectory (or position) can be estimated. However, IMU-based PDR relies too much on a pedestrian’s walking pattern having a uniform speed and is sensitive to the change in a pedestrian’s walking gestures in practical situations. There are two main reasons causing unacceptable accumulating errors that grow with the path length: (1) gravity influence; (2) the drift problem. To minimize this error, plenty of attempts have been made, but mostly by leveraging additional infrastructures.

### 3.3. Basics of Doppler Effect

As an observer moves relative to a wave source, the frequency of the wave changes, which is called the Doppler effect [17]. In our case, due to the relative motion between two pedestrians, the sub-ultrasonic emitted by one smartphone takes slightly less or more time to reach the other smartphone, which mainly depends on whether the two pedestrians are moving closely or far away. The frequency offset is determined by the relative velocity between two pedestrians (as shown in Equation (1)).

(1) $$\Delta f=\frac{\Delta v}{c}\times f_{e}$$

where Δv represents the relative velocity between two pedestrians. *c* is the speed of acoustic traveling in air, which is 340 m/s. $f_{e}$ denotes the emitted frequency of acoustic, and frequency offset Δf can also be calculated through $\Delta f=f_{r}-f_{e}$ once we obtain the received frequency $f_{r}$.

### 3.4. Key Issues

In order to realize an infrastructure-free indoor pedestrian tracking approach with a smartphone sub-ultrasonic-based Doppler effect, we have to deal with three key issues listed as follows:

**Key Issue 1**. In our scenario, iIPT adjusts a relatively less reliable pedestrian speed to a more reliable speed of a passing by “enhancer” based on the sub-ultrasonic Doppler effect. In terms of the acceleration data sensed by accelerometers, how to define a proper metric to characterize the reliability of the pedestrian speed provided by smartphone IMU is investigated.

**Key Issue 2**. Little research has been done on the sub-ultrasonic Doppler effect between two smartphones, so other relevant methods cannot be directly leveraged in our scenario. Due to the variety of commercial smartphones (Android or iOS), their ability to emit and receive sub-ultrasonic may be different from each other. It is a key issue to identify the applicable sub-ultrasonic frequency range and transmission distance for the Doppler effect while taking battery consumption into consideration.

**Key Issue 3**. When two pedestrians are moving face-to-face, their walking speed is unknown and the speed value calculated through the Doppler effect is relative rather than absolute. Without a baseline, how to enhance the user’s walking speed to finally improve the pedestrian tracking accuracy becomes a key issue.

This section presents a big picture for iIPT, including the elaboration of our basic idea, IMU-based PDR, the Doppler effect, and three key issues. We detail our core method in Section 4.

## 4. Proposed Approach

Starting with the problem statement, this section elaborates our proposed method by leveraging both experimental and theoretical analysis.

### 4.1. Problem Statement

There are only two pedestrians in our encounter model: one is the “user” and the other is the “encounter”. To better illustrate our proposed method, we firstly make several definitions as follows:

**Definition 1.**  ***(DopE Direction).** DopE direction is defined as the sub-ultrasonic propagating direction, which is along the straight line from the sub-ultrasonic emitter to the receiver.*

**Definition 2.**  ***(DopE Speed).** DopE speed Δv is defined as the relative speed in the DopE direction between user and encounter, calculated by the sub-ultrasonic Doppler effect:*

(2) $$\Delta v=c\times \frac{f_{r}-f_{e}}{f_{e}}$$

*where c is the speed of sub-ultrasonic traveling in air, which is 340 m/s, $f_{e}$ denotes the emitted frequency of sub-ultrasonic, and $f_{r}$ represents the received frequency.*

**Definition 3.**  ***(DopE Angle).** The DopE angle θ is defined as the angle between the pedestrian’s walking direction and the DopE direction:*

(3) $$\sin\theta =\frac{L}{D}$$

**Figure 4:**
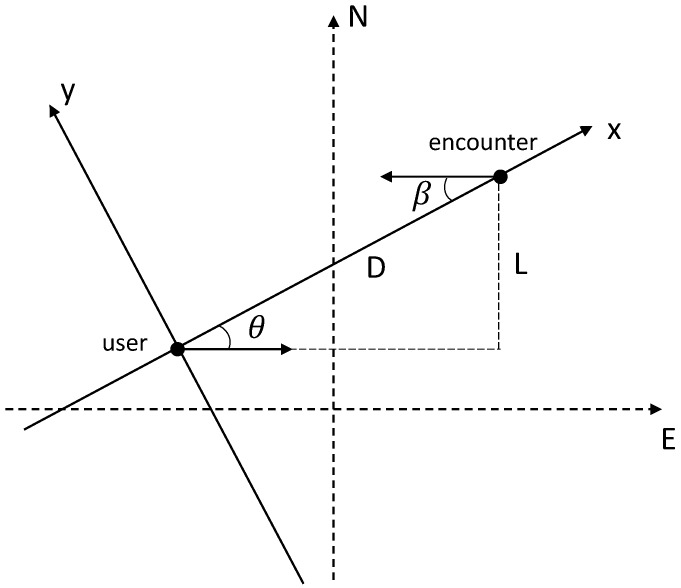
Pedestrian encounter model for the calculation of the DopE angle and the DopE speed.

*where L represents the width of the hallway, and D represents the shortest distance between user and encounter (shown in Figure 4).*

We assume that the two pedestrians’ walking direction is parallel in most cases of indoor hallway walking situations. Therefore, θ=β.

**Definition 4.**  ***(Pedestrian Speed Reliability).** Pedestrian speed reliability ρ is defined as the variance value of acceleration sensed by smartphone IMU.*

The reliability is lower when the pedestrian is walking arbitrarily. Therefore, we consider Δρ as a threshold and utilize the encounter’s speed as a benchmark when Δρ is below a certain value.

(4) $$\Delta \rho =\rho_{encounter}-\rho_{user}.$$

### 4.2. Sub-Ultrasonic Doppler Identification

Due to the variety of commercial smartphones (Android or iOS), their ability to emit and receive sub-ultrasonic are different from each other. In order to obtain the sub-ultrasonic frequency range (especially the upper limit) and a reliable communication distance, we carried out a few experiments in a practical situation.

#### 4.2.1. Sub-Ultrasonic Frequency

**Figure 5:**
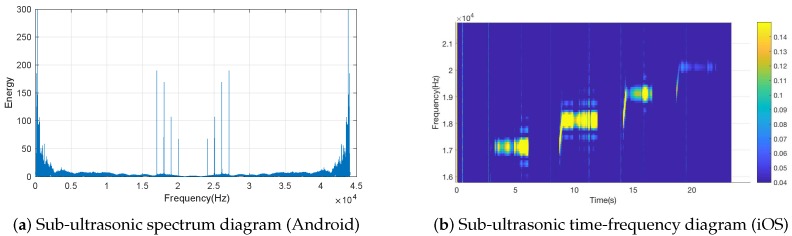
Identification of smartphone-based sub-ultrasonic frequency after Fourier transformation.

Scientifically, ultrasonic is defined as sound waves with frequencies higher than the upper audible limit of human hearing (20 kHz). According to [15], commercial smartphones can emit and receive 17–22 kHz acoustic, but no further description is presented in these studies. Moreover, these studies are implemented in a phone-to-infrastructure situation, which is different from a phone-to-phone scenario. To find more information, we carried out an experiment on an acoustic frequency range under a phone-to-phone situation. By leveraging both Android and iOS smartphones, we continuously emitted and received acoustic from 17 to 22 kHz, and the result is depicted in Figure 5, showing that both Android and iOS smartphones have the ability to emit and receive acoustic from 17 to 20 kHz under a phone-to-phone situation.

#### 4.2.2. Sub-Ultrasonic Transmission Distance

According to [29], the sub-ultrasonic transmission distance under a phone-to-infrastructure situation is at least 20 m (even higher, depending on the infrastructure type), and usually the smartphone works as a receiver and the infrastructure works as an emitter so that a longer transmission distance is guaranteed. In this paper, both the emitter and receiver are smartphones, leading to new requirements for the study on phone-to-phone sub-ultrasonic scenarios. The sub-ultrasonic transmission distance is mainly determined by a smartphone’s volume level. The higher the volume we utilize, the longer the distance it will transmit, but it will also generate a more noisy sound at the same time.

**Figure 6:**
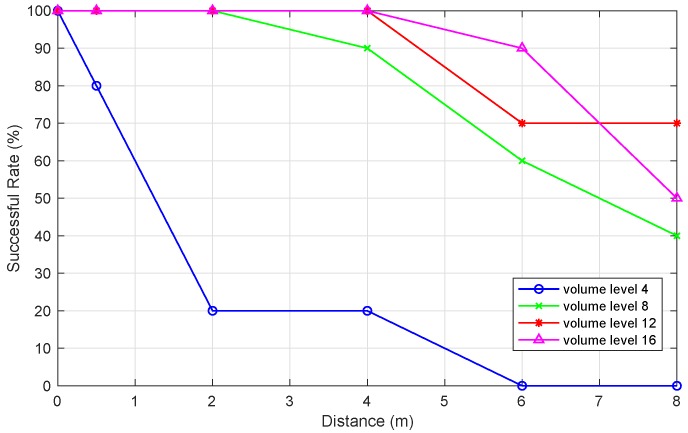
The relationship between the success rate of receiving sub-ultrasonic by smartphones and sub-ultrasonic transmission distance with different smartphone volumes.

We can see from Figure 6 that a smartphone sub-ultrasonic signal can be unreliably detected by another phone from 8 m away. Moreover, as distance increases, the success rate of receiving sub-ultrasonic with different volumes drops. To be more detailed, 25% of the volume of sub-ultrasonic performs terribly, even in a short distance. Within 4 m, the success rate of volume level 12 and level 16 are both 100%, and as the distance increases, a higher volume sub-ultrasonic transmits more reliably. However, when the distance reaches 8 m, volume level 16 performs better than level 8 because of its higher power, but its performance is worse than level 12 due to the more noisy sound generated. Taking the density of indoor pedestrians into account, this sub-ultrasonic transmission distance analysis provides a fundamental reference for the sub-ultrasonic Doppler effect in the indoor pedestrian encounter scenario.

#### 4.2.3. Pedestrian Moving Detection

**Figure 7:**
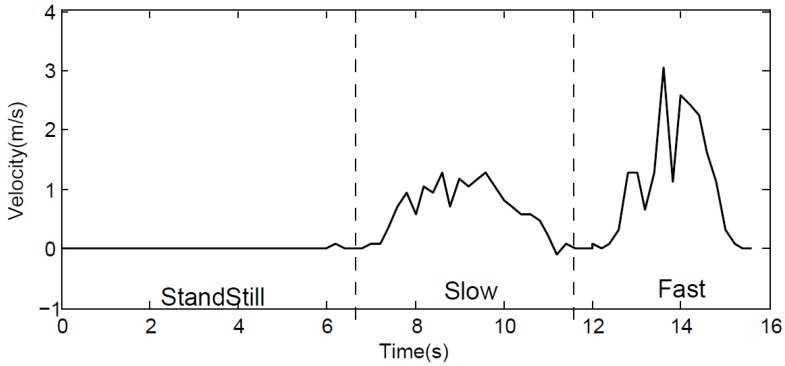
Pedestrian walking detection by utilizing the Doppler effect with 19.5 kHz sub-ultrasonic.

In most cases, the Doppler effect is utilized to fulfill high precision movement detection in a short distance [15,30]. To demonstrate the feasibility of leveraging the Doppler effect to obtain an encounter profile, we design a standstill-slow-faster mode experiment based on our experiment analysis above. At first, the pedestrian begins to leverage the smartphone to emit 19.5 kHz sub-ultrasonic but has to remain standstill for a while. Then they start to walk slowly towards the other phone and afterwards walk fast. We store the received audio data in a PCM file, we cut the audio files into several parts, which are 0.2 s long, and the frequency of each audio file is calculated by Fourier transform one by one. In this experiment, the length of every audio file is a key parameter. We hope to minimize the length of each separated files as much as possible so that every frequency calculated by Fourier can be regarded as the instantaneous frequency. However, at a fixed sampling rate, while reducing the cutting interval, the accuracy of the frequency will be decreased. Correspondingly, the accuracy of velocity will be cut down. After our repeated experiments, we discover that 0.2 s is a decent choice for balancing the accuracy and instantaneity, which achieves a 0.1 m/s velocity error. However, for the accuracy deduced by a 0.1 s file, the error will be above 0.3 m/s. As shown in Figure 7, the frequency of the received sub-ultrasonic becomes higher after the pedestrian speeds up. In a word, the Doppler effect is able to detect pedestrian walking in an indoor pedestrian encountering scenario.

#### 4.2.4. Robot Car Moving Detection

**Figure 8:**
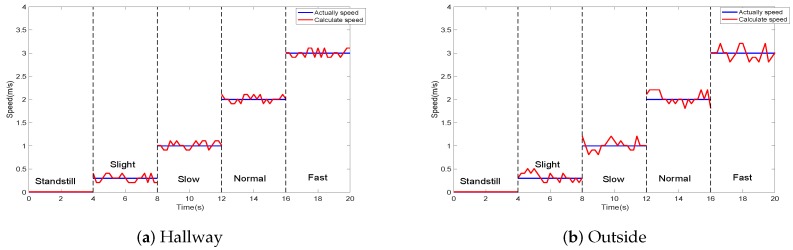
Comparisons of the measured speed and the actual speed.

**Figure 9:**
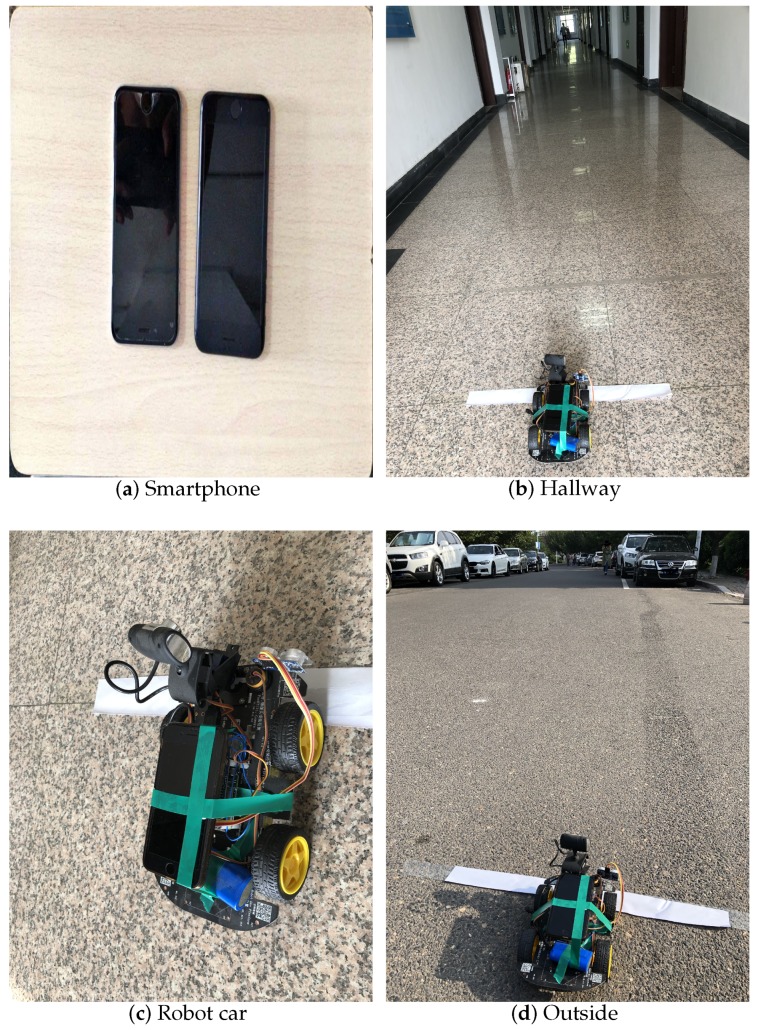
Indoor experiment environment.

Since we cannot measure the exact instantaneous speed of the volunteer at a certain instant, we designed a robot car (shown in Figure 9c). We fixed the mobile phone on the robot car, which was controlled by Arduino. It can be programmed to move forward at an accurate speeds of 0.3, 1, 2, and 3 m/s. We carried out experiments in two experimental environments, that is, in a hallway (shown in Figure 9b) and outside Figure 9b. In the standstill, slight, slow, normal, and fast moving states of the car, we compared the real speed and the measured speed of the robot car after the robot car’s speed was stable. The result is shown in Figure 8. Compared with the indoor environment, the outdoor environment measurement results are more unstable.

### 4.3. Walking Speed Enhancement

In this paper, we assume that the two pedestrians’ walking direction is parallel in most cases of the indoor hallway walking situations. Therefore, the relative angle between the user’s (or the encounter’s) walking direction and the DopE direction is calculated by the triangle we built. Afterwards, we leveraged the encounter’s walking speed and the DopE speed to finally obtain the user’s speed. More detail is shown in Algorithm 1. In the end, based on the DopE speed Δv, the DopE angle θ, and the encounter’s walking speed $v_{encounter}$, we built a mathematical model for computing the user’s walking speed, which is shown in Equation (5).

(5) $$v_{user}=\frac{\Delta v-v_{encounter}\times \sqrt{1-{\sin\theta }^{2}}}{\cos\theta }.$$

**Algorithm 1:** Walking speed enhancement. **Input:** hallway width *L*           acoustic speed *c* **Output:** User walking speed $v_{user}$
**1** Emit $f_{e}$ kHz sub-ultrasonic;**2** Receive $f_{r}$ kHz sub-ultrasonic;**3** $\Delta v=c\times \frac{f_{r}-f_{e}}{f_{e}}$;**4** D← sub-ultrasonic TDOA method;**5** $\sin\theta =\frac{L}{D}$;**6** The velocity component of $v_{encounter}$ on DopE direction;**7** $v_{encounter}^{\prime }=v_{encounter}\times \sqrt{1-{\sin\theta }^{2}}$;**8** The velocity component of $v_{user}$ on DopE direction;**9** $v_{user}^{\prime }=\Delta v-v_{encounter}^{\prime }$;**10** return $v_{user}$;

The proposed method is presented in this section. In next section, we implement a few more experiments to demonstrate that iIPT can largely improve the tracking accuracy and decrease the average error compared with a conventional IMU-based PDR method.

## 5. Performance Evaluation

In order to demonstrate the feasibility and accuracy of iIPT, we implemented several experiments in a teaching building on campus. By using certain commercial smartphones, we verified the Doppler effect’s accuracy of a pedestrian’s moving detection and compared the iIPT performance with that of a conventional IMU-based PDR method. At last, we illustrate our research on battery consumption.

### 5.1. Indoor Environment Description

**Smartphone**. In terms of our experiment result in Section 4, commercial smartphones have a similar ability to emit and receive sub-ultrasonic. Taking our own situation into account, we utilized an iPhone 7 to implement our experiment and leveraged pulse code modulation (PCM) files to store and analyze the sub-ultrasonic data. Besides, the format of the PCM is a mono-channel, little endian. The sampling frequency is 96,000 Hz, and the quantization accuracy is 16 bit.

**Hallway environment**. The experiment site is a hallway in our college building that is 2 m wide, which is shown in Figure 9b. Because the temperature is up to 31 ${}^{\circ }$C, we adopted 349 m/s as the sub-ultrasonic velocity. We measured the pedestrian and the robot car in the same hallway environment.

**Robot car**. We fixed the mobile phone on this robot car, which is shown in Figure 9c. The robot car is controlled by Arduino and has high speed accuracy.

**Outside environment**. The outside environment is on the road, which is shown in Figure 9d.

### 5.2. Tracking Accuracy

**Figure 10:**
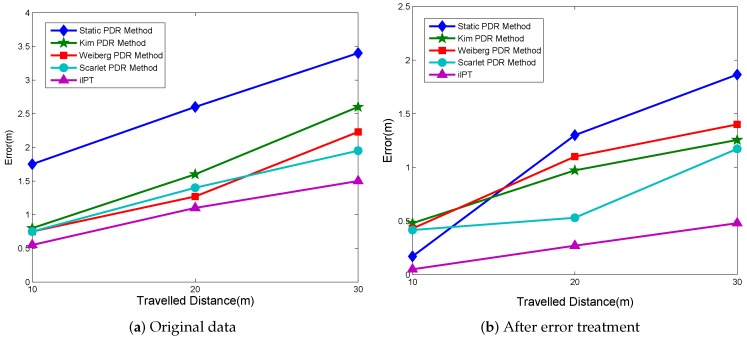
Comparison of tracking accuracy between PDR and iIPT.

To verify iIPT’s capability of improving pedestrian tracking accuracy when the pedestrian walks arbitrarily, we implemented a set of 6 m pedestrian encountering plots, utilized iIPT to estimate the walking distance, and compared the tracking result with IMU-based PDR methods. Four different kinds of PDR methods were selected as the contract: 1. the Stastic method; 2. the Kim method; 3. the Weiberg method; 4. the Scarlet method [31]. In general, the IMU-based PDR experiment will take a traveled distance of over 10 m. However, as shown in Figure 6, the ultrasonic Doppler effect’s performance is reliable only within 6 m because of the characteristic of its fast energy attenuation. Therefore, to extend the traveled distance of ilPT to make it comparable to the PDR method, the number of experiments was increased, and we accumulated errors that can be regarded as one long distance experiment. We compared the total average error of our tracking method with these four PDR methods mentioned above. In order to eliminate the accidental error, every group of experiment for different methods was constructed 20 times. The average experiment result before subtracting the systematic error is shown in Figure 10a. It is obvious that the accuracy of the static PDR method is not good enough to satisfy the short distance tracking situation. Besides, the accumulative distance bias of the Kim, Weiberg, and Scarlet PDR methods is 0.75 m at 10 m and increases up to over 1.25 and 1.9 m at 20 and 30 m, respectively, while iIPT maintains a stable bias around 0.5 m every 10 m. In addition, it is inevitable that the experimental data always contain a systematic error due to various factors. Therefore, we supplement an experiment that removed the systematic error. In the course of our experiment, we found that the frequency of ultrasonic smartphones received always has a 0.5 Hz fixed deviation, possibly caused by the hardware. Similarly, the PDR methods have a different systematic error with a certain traveled distance. The experiment result after subtracting the systematic error is shown as Figure 10b. The iIPT obviously provides a smaller error compared to the four PDR methods. The average error can be controlled within 2%.

**Figure 11:**
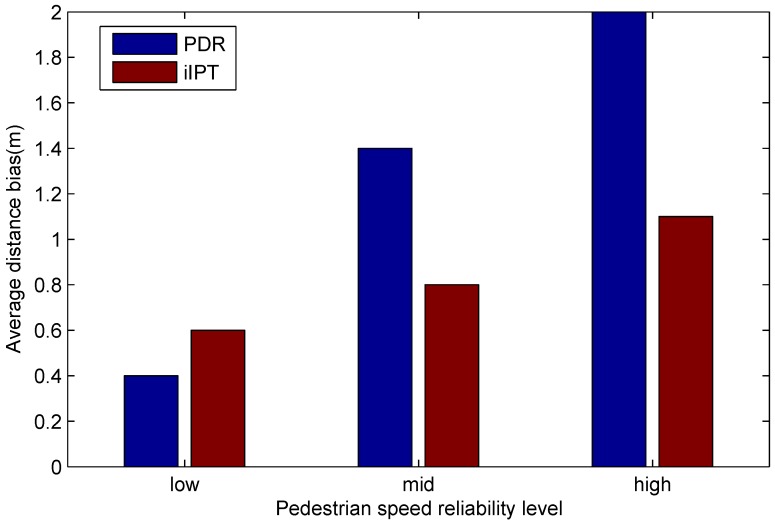
Comparison of tracking distance bias between PDR and iIPT under different pedestrian speed reliability levels.

In addition, we classified the pedestrian speed reliability ρ into three levels: low (0<=ρ<0.005), mid (0.005<=ρ<0.01), and high (0.01<=ρ). Tracking distance bias changes with different levels, and the comparison between iIPT and PDR is shown in Figure 11. We can see from the experiment result that the average distance bias of PDR increases severely with a higher ρ. On the contrary, iIPT is less sensitive to ρ, although the bias still slightly increases. In a word, iIPT performs better than PDR at tracking a pedestrian with arbitrary walking.

### 5.3. Battery Consumption

**Figure 12:**
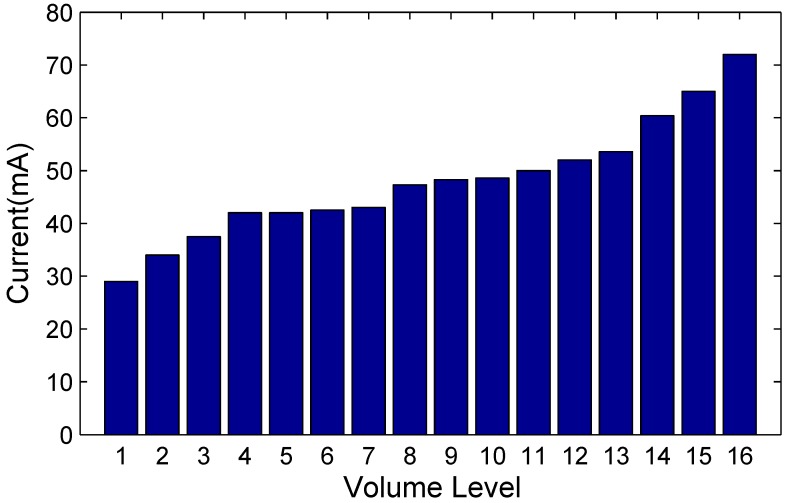
Smartphone’s battery consumption when emitting 19.5 kHz sub-ultrasonic with different volume levels.

For iIPT, the smartphone’s battery consumption is mainly determined by a sub-ultrasonic emitting volume level, which has a vital influence on sub-ultrasonic transmission distance. As depicted in Figure 12, it is obvious that the battery consumption increases with a higher volume level. From level 1 to level 4, battery consumption increases by 50%, and as the level increases, it increases smoothly up to level 12. From level 12 to level 16 (max volume), the battery consumption increases severely again. Moreover, taking transmission distance into account at the same time, we consider a volume around level 12 to be suitable for the sub-ultrasonic Doppler effect in an indoor environment.

According to the experiment result, iIPT performs better than the IMU-based tracking method under arbitrary walking circumstance. However, there are still several unsettled problems, and we have a brief discussion of those problems in Section 6.

## 6. Discussion

**One-on-N encounter model**. In our scenario, we only consider a one-on-one pedestrian encounter model so that there is only one user and one encounter. However, in actual situations, there will be multiple encounters around the user, increasing the complexity of the Doppler effect analysis and the amount of ultrasonic reflection. A one-on-N pedestrian encounter model is essential for the multiple encounter scenario, but how to select proper encounters to build the local model becomes a huge problem. Moreover, the one-on-N model causes serious multi-path problems and increases the complexity of analyzing ultrasonic signals due to additional noise.

**Relative angle calculation**. In this paper, we set our indoor background as a hallway, and the relative angle between the user (or the encounter) and the DopE direction was obtained by calculating a triangle, which was based on the assumption that the width of the hallway can be known ahead of time. In addition, the relative angle between the walking direction of the user and the encounter becomes larger when the two pedestrians are walking more closely to each other. Due to the complex indoor environment, how to extend iIPT to deal with more indoor situations is another key problem.

**Encounter benchmark selection**. We consider that the variance of acceleration sensed by smartphones becomes higher when pedestrians walk arbitrarily, which represents the reliability of the pedestrian walking speed from IMUs. Therefore, we chose an encounter with lower variance to modify the user’s speed. However, it is still difficult to find metrics for measuring pedestrian speed reliability, which should be defined more specifically so that iIPT will be able to deal with different indoor pedestrian encounter situations.

## 7. Conclusions

In this paper, we propose an infrastructure-free pedestrian tracking approach by combining smartphone-based sub-ultrasonic and IMU techniques. A metric called pedestrian speed reliability is presented to characterize the reliability of the pedestrian speed provided by smartphone IMU. We adjust a relatively less reliable pedestrian speed to a more reliable speed of a passing by ”enhancer“ based on the sub-ultrasonic Doppler effect, thus changing encountered pedestrians from an ”obstacle“ into an ”enhancer“. Extensive real-world experiments in indoor scenarios have been conducted to verify the feasibility of realizing the acoustic Doppler effect between smartphones and to identify the applicable sub-ultrasonic frequency range and transmission distance while reducing battery consumption. The experiment results demonstrate that iIPT can largely improve the tracking accuracy and decrease the average error compared with a conventional IMU-based method. For further research, we plan to extend the encounter model to multiple situations and use crowd sensing technology to solve the encounter selection problem.

## Figures and Tables

**Table 1:** Frequency range of acoustic from commercial smartphones.

Smartphone	OS	Frequency	Reference
HTC G1	Android		
HTC Hero	Android	17–22 kHz	Ref [9]
HiPhone 3GS	ios		
Nokia 6210 Navigator	Android		
Samsung Galaxy S5	Android	17–23 kHz	Ref [7]
HiPhone 6S	ios		
Samsung Galaxy S7	Android		Our research
iPhone 7	ios	17–20 kHz	(phone-to-phone)
iPhone x	ios		
